# A Comprehensive Subcellular Proteomic Survey of *Salmonella* Grown under Phagosome-Mimicking versus Standard Laboratory Conditions

**DOI:** 10.1155/2012/123076

**Published:** 2012-07-25

**Authors:** Roslyn N. Brown, James A. Sanford, Jea H. Park, Brooke L. Deatherage, Boyd L. Champion, Richard D. Smith, Fred Heffron, Joshua N. Adkins

**Affiliations:** ^1^Biological Sciences Division, Pacific Northwest National Laboratory, 902 Battelle Boulevard, Richland, WA 99352, USA; ^2^Biomedical Sciences Graduate Program, University of California San Diego, 9500 Gilman Dive, La Jolla, CA 92063, USA; ^3^Department of Molecular Microbiology and Immunology, Oregon Health and Science University, 3181 SW Sam Jackson Park Road, Portland, OR 97239, USA

## Abstract

Towards developing a systems-level pathobiological understanding of *Salmonella enterica*, we performed a subcellular proteomic analysis of this pathogen grown under standard laboratory and phagosome-mimicking conditions *in vitro*. Analysis of proteins from cytoplasmic, inner membrane, periplasmic, and outer membrane fractions yielded coverage of 25% of the theoretical proteome. Confident subcellular location could be assigned to over 1000 proteins, with good agreement between experimentally observed location and predicted/known protein properties. Comparison of protein location under the different environmental conditions provided insight into dynamic protein localization and possible moonlighting (multiple function) activities. Notable examples of dynamic localization were the response regulators of two-component regulatory systems (e.g., ArcB and PhoQ). The DNA-binding protein Dps that is generally regarded as cytoplasmic was significantly enriched in the outer membrane for all growth conditions examined, suggestive of moonlighting activities. These observations imply the existence of unknown transport mechanisms and novel functions for a subset of *Salmonella* proteins. Overall, this work provides a catalog of experimentally verified subcellular protein locations for *Salmonella* and a framework for further investigations using computational modeling.

## 1. Introduction

The pursuit of a systems-level understanding of bacterial physiology requires not only knowledge about the identity, function, and relative abundance of proteins, but also insight into the subcellular localization of these proteins. Subcellular protein localization is linked to protein function, potential protein-protein interactions, and to interactions between a cell and its exterior environment. The observation of proteins in unexpected cellular compartments gives clues about the presence of possible alternate functions. Hence, there is a growing appreciation for the presence of bacterial “moonlighting proteins,” that is, those proteins that have a secondary function depending on subcellular location [1–3]. Experimentally verified localization also provides a foundation for describing proteins that are “hypothetical,” uncharacterized, or that contain domains of unknown function. Furthermore, with the increasing use of systems biology approaches, including genome-scale models of metabolism [4] and regulation to study microbial functions, experimentally founded protein localization on a global scale is necessary to produce more accurate model constraints.

Subcellular proteomics has emerged as a powerful tool for large-scale profiling of protein subcellular location [5–9]. Unlike traditional Western blot or high-resolution microscopy methods that rely on the use of antibodies or molecular tags to identify individual proteins, proteomic methods enable high-throughput, unbiased, and large-scale identification of the protein complement of subcellular fractions [5, 6, 10]. Moreover, interrogation of the subcellular proteome under different growth or environmental conditions allows for the investigation of changes in protein abundance and possibly protein location.

Subcellular proteomic analysis of bacterial pathogens holds promise for identifying novel virulence determinants and potential therapeutic targets [11–13]. For Gram-negative pathogens such as *Salmonella enterica*, each of the four main protein-containing compartments—the outer and inner membranes, periplasm, and cytoplasm—is a potential source of virulence determinants. Outer membrane/cell surface proteins mediate adhesion, cell-cell communication, immune evasion, sequestration, transport (including antibiotic efflux), and secretion, whereas inner membrane proteins accomplish transport and assembly of complex structures, such as flagella and secretion apparati. Periplasmic proteins sense and respond to the host environment, and cytoplasmic proteins include secretion substrates, chaperones, and housekeeping proteins important in maintaining the pathogenic lifestyle. Comprehensive characterization of these subcellular fractions can provide insight into the potential for virulence-related interactions with the host as well as fundamental information on the subcellular architecture of this organism.

Our present goals were twofold: (1) to survey the localization of proteins in *Salmonella* cells as a reference of protein localization in this bacterium and (2) to observe changes in protein abundance or location upon growth under phagosome-mimicking conditions relative to standard laboratory conditions to generate new biological insights, as well as improved data for computational modeling. Towards this end, cytoplasmic (CYT), inner membrane (IM), periplasmic (PERI), and outer membrane (OM) fractions were analyzed using liquid chromatography-tandem mass spectrometry (LC-MS/MS). We did not analyze the secretome as we recently completed an extensive analysis of the proteins secreted by *Salmonella* under phagosome-mimicking conditions [14]. In the present study, over 25% of the theoretical *Salmonella* proteome was represented, and confident assignment of subcellular locations was achieved for most proteins. In addition, we assigned subcellular-level localization to the response of the bacteria to growth under conditions that mimic the host macrophage intracellular environment. This study represents the most comprehensive global survey of subcellular localization in *Salmonella* to date and affords a resource to others interested in protein location, improving location predictions and systems computational models.

## 2. Methods

### 2.1. Rationale for Media and Strains Used in This Study

Growth to mid-logarithmic phase in Luria-Bertani broth represents a standard laboratory growth condition in this study and is noninducing for *Salmonella* pathogenicity island 2 (SPI-2) gene expression [15]. Growth of *Salmonella *in defined, acidic media with low concentrations of phosphate and magnesium induces expression of SPI-2 genes that are required for intracellular survival and replication [15–19]. mLPM has been shown to induce expression and secretion of SPI2-related virulence factors [14] and was used in this study to mimic the environment of a macrophage phagosome.

We previously identified flagellin (especially FliC) as one of the most abundant proteins secreted by *Salmonella* into culture media [14] and also in cell envelope fractions (Supplemental Table 1, supplementary material available online at doi: no# 10.1155/2012/123076). *Salmonella* flagellins are downregulated during the intracellular stage of infection, and SPI-2-expressing bacteria are not motile [20]. Since flagella are not relevant to the stage of infection we intended to mimic, we deleted flagellin genes *fliC *and *fljB* from wildtype *Salmonella enterica *serovar Typhimurium (*S*. *Typhimurium*) ATCC 14028 in an attempt to achieve better sensitivity by depleting these abundant proteins.

### 2.2. Bacterial Strains, Media, and Chemicals

Bacteria were maintained in LB broth (Difco, Franklin Lakes, NJ, USA) or on LB plates. Unless otherwise noted, components of mLPM [14] and other chemicals were purchased from Sigma (St. Louis, MO, USA). Protein concentrations were determined by bicinchoninic acid (BCA) assay (Pierce, Rockford, IL, USA) using bovine serum albumin as standards. Trypsin used for protein digestions was purchased from Promega (Madison, WI, USA).

### 2.3. Deletion of Flagellin Genes

In an attempt to achieve better sensitivity by depleting a nonessential abundant protein (Supplemental Table 1), a double-flagellin mutant (Δ*fliC*Δ*fljB*) was created using *λ* Red recombination [21]. *fliC *was deleted using oligos FliC P1: AGCCCAATAACATCAAGTTGTAATTGATAAGGAAAAGATCGTGTAGGCTGGAGCTGCTTC and FliC P2: CCTTGATTGTGTACCACGTGTCGGTGAATCAATCGCCGGACATATGAATATCCTCCTTAG.

For deleting *fljB*, oligos FljB P1: GATTTTCTCCTTTACATCAGATAAGGAAGAATTTTAGTCGGTGTAGGCTGGAGCTGCTTC and FljB P2: CTCGCCCGTAGGAAATATCATTTACAGCCATACATTCCATCATATGAATATCCTCCTTAG were used. Underlined portions of the above oligos represent pKD4 sequences. Insertion of the kanamycin resistance cassette was confirmed using oligos FliC test1: AATGATGAAATTGAAGCCAT and K1: CAGTCATAGCCGAATAGCCT for *fliC* and using FljB test1: AACGCCACCAGGTTTTTCAC and K1 for *fljB*. The kanamycin resistance gene was removed using pCP20 as previously described [21]. The flagellin mutant was tested for lack of motility, compared to the wildtype, using 0.4% agar plates.

### 2.4. Subcellular Fractionation

Overnight starter cultures of WT and the Δ*fliC*Δ*fljB* mutant were grown in LB broth at 37°C with shaking at 200 rpm. The cultures were diluted 1 : 100 into LB and grown to mid-log phase (OD600 ~ 0.6) for the “LB-log” condition or diluted 1 : 10 into mLPM and grown for 4 or 20 h for “LPM4” and “LPM20,” respectively.

The cell fractionation protocol was adapted from that described by Brown et al. [9]. Unless otherwise noted, centrifugation steps were performed at 4°C. Cells were collected via centrifugation (10,000 ×g, 10 min) and washed with 10 mL of 50 mM Tris-HCl (pH 8.0). PERI fractions were generated by suspending cell pellets in 10 mL spheroplasting buffer (50 mM Tris-HCl, pH 8, 250 mM sucrose, 2.5 mM EDTA) and incubating at room temperature for 5 min, after which they were centrifuged at 11,500 ×g for 10 min. Pellets were then suspended in 1.3 mL cold 5 mM MgSO_4_ and kept on ice for 10 min with occasional mixing. After centrifugation (11,500 ×g, 10 min), the supernatant was retained as the soluble PERI fraction, while the pelleted spheroplasts were suspended in 1.0 mL 20 mM NaH_2_PO_4_.

Half of the spheroplasts from each condition were then used to perform fractionation into CYT, IM, and OM fractions. The volumes were adjusted to 3.0 mL in 20 mM NaH_2_PO_4_ and lysed by passing three times through a prechilled French Press (8,000 PSI). Cell lysate suspensions were adjusted to 10 mL using 20 mM NaH_2_PO_4_ and centrifuged at 5,000 ×g for 30 min to pellet unbroken cells. Supernatants were then centrifuged at 45,000 ×g for 60 min to separate the soluble CYT fraction from the crude membrane pellet. The CYT fractions were centrifuged again to remove residual membrane contaminants. After tubes containing membrane pellets were inverted to dry, the pellets were suspended in 10 mL 20 mM NaH_2_PO_4_ containing 0.5% Sarkosyl and shaken at 200 rpm for 30 min at room temperature. This mixture was then centrifuged at 45,000 ×g for 60 min to pellet the OM fraction, and the supernatant containing the IM fraction was removed. OM fractions were washed once by suspending in 5 mL NaH_2_PO_4_ and repeating the centrifugation.

### 2.5. Tryptic Digests

Tryptic digests of the soluble CYT and PERI fractions were prepared as follows. To 75 *μ*g of protein from each sample, urea and DTT were added to final concentrations of 7 M and 5 mM, respectively, followed by incubation at 60°C for 30 min. Samples were then diluted 7-fold with 100 mM NH_4_HCO_3_, and CaCl_2_ was added to a final concentration of 1 mM. Trypsin was then added in a 1 : 50 trypsin : protein ratio, and digestions were performed at 37°C with shaking at 600 rpm for 3 hours. Following digestion, samples were cleaned using 1 mL, 50 mg Discovery DSC-18 solid phase extraction (SPE) columns (Supelco, St. Louis, MO, USA). Briefly, each column was conditioned with methanol and then rinsed with 0.1% TFA in water. Digested samples were run through the columns under vacuum and rinsed with 95 : 5 H_2_O : ACN with 0.1% TFA. Excess liquid was removed from the columns, and peptides were eluted using 80 : 20 ACN : H_2_O containing 0.1% TFA. Peptides were collected and concentrated using a SpeedVac (Thermo-Savant) to a final volume of 50–100 *μ*L, after which final peptide concentrations were determined by BCA protein assay.

Tryptic digests of the insoluble IM and OM fractions were prepared as follows. To 75 *μ*g of protein from each sample, urea, DTT, and CHAPS were added to final concentrations of 7 M, 10 mM, and 1%, respectively, followed by incubation at 60°C for 30 min. Samples were then diluted 7-fold with 100 mM NH_4_HCO_3_, and CaCl_2_ was added to a final concentration of 1 mM. Digestion was performed as described for the soluble fractions. Digested samples were then cleaned using 1 mL, 50 mg Discovery SCX strong cation exchange SPE columns (Supelco, St. Louis, MO, USA). Briefly, columns were conditioned with methanol and then rinsed in varying sequences and amounts of 10 mM ammonium formate in 25% ACN (pH 3.0), 500 mM ammonium formate in 25% ACN (pH 6.8), and nanopure water. Peptide samples were acidified to pH < 4 with formic acid, centrifuged at 10,000 ×g for 5 min, applied to the columns, and washed with 10 mM ammonium formate in 25% ACN (pH 3.0). Peptides were eluted using 80 : 15 : 5 MeOH : H_2_O : NH_4_OH and concentrated to a final volume of 50–100 *μ*L using a SpeedVac. Final peptide concentrations were calculated by BCA protein assay.

### 2.6. SDS-PAGE

For visualization of the protein fractions, 5 *μ*g of each protein sample was suspended in NuPAGE LDS sample buffer (Invitrogen, Carlsbad, CA, USA), heated at 70°C for 10 min, and resolved on NuPAGE Novex 4–12% Bis-Tris gradient gels (Invitrogen). Gels were run at a constant voltage of 200 V for 35 min and subsequently stained with GelCode Blue stain (Pierce) to observe protein profiles.

### 2.7. Capillary LC-MS/MS Analysis

The high-performance liquid chromatography (HPLC) system and method used for nanocapillary liquid chromatography have been described in detail elsewhere [19, 22]. Analysis was performed using an LTQ-Orbitrap mass spectrometer (Thermo Fisher Scientific, San Jose, CA, USA) with electrospray ionization. The HPLC column was coupled to the mass spectrometer using an in-house manufactured interface. The heated capillary temperature and spray voltage were 200°C and 2.2 kV, respectively. Data acquisition began 20 min after the sample was injected and continued for 100 min over an *m/z *range of 400–2000. For each cycle, the six most abundant ions from MS analysis were selected for MS/MS analysis, using a collision energy setting of 35 eV. A dynamic exclusion time of 60 s was used to discriminate against previously analyzed ions. All subcellular fractions from the Δ*fliC*Δ*fljB* mutant were analyzed in addition to the PERI of the WT (Supplemental Table 2) to ensure that the loss of flagellins did not alter periplasmic proteome expression. Each sample was analyzed in triplicate.

### 2.8. Data Analysis

Peptides were identified by using SEQUEST to search the mass spectra from LC-MS/MS analyses. These searches were performed using the annotated *S*. *Typhimurium* 14028 FASTA file, containing 5590 protein sequences [23]. Porcine trypsin protein sequences were included in the search to detect trypsin autocleavage contaminants. The SEQUEST parameter file contained no modifications to amino acid residues and a mass error window of 3 *m/z *units for precursor mass and 0 *m/z *units for fragmentation mass. The searches allowed for all possible peptide termini, that is, not limited by tryptic terminus state. Results were filtered using the MS-Generating Function [24], a software tool that assigns *P* values (spectral probabilities) to spectral interpretations. The prescribed spectral probability cutoff (1E^−10^) was used. This corresponded to a false-positive rate of 0.88% at the unique peptide level and 0.16% and the spectrum level using a traditional decoy approach, that is, searching against a reversed FASTA database [25].

The number of peptide observations from each protein (spectral count) was used as a measure of relative abundance. Multiple charge states of a single peptide were considered as individual observations, as were the same peptides detected in different mass spectral analyses. Similar approaches for quantitation have been described previously [9, 14, 19, 26]. A protein was considered present in a sample (subcellular fraction) only if observed in at least 2 of 3 technical replicates, and means of triplicate samples were adjusted to zero if this rule was not satisfied.

Statistical analyses were performed using Microsoft Excel and R (http://www.r-project.org/). K-means clustering and construction of heat maps were done using OmniViz 6.0.

## 3. Results

### 3.1. Protein Identification in *Salmonella* Subcellular Fractions

**Figure 1:**
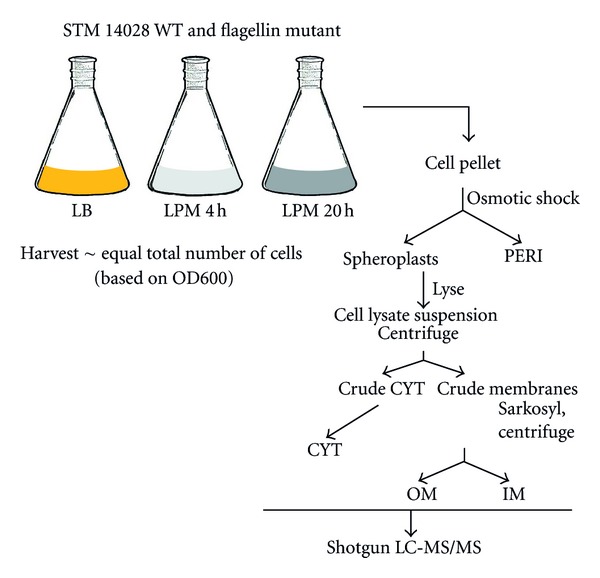
Experimental workflow. A fractionation scheme based on differential centrifugation and Sarkosyl solubilization of membranes was combined with spheroplasting to obtain PERI, CYT, OM, and IM samples from *S. Typhimurium* strain 14028. Subcellular fractions were further processed prior to high-resolution LC-MS/MS analysis.

To survey the localization of proteins in *Salmonella* cells as a reference of protein localization and to observe changes in protein abundance upon growth under phagosome-mimicking conditions relative to standard laboratory conditions, *S. Typhimurium* 14028 flagellin mutant (see Section 2 for rationale) was grown in Luria-Bertani broth (LB) to log phase or in a low-phosphate, low-magnesium, low-pH minimal medium (LPM) for 4 or 20 h. Subcellular fractionation based on osmotic shock, differential centrifugation, and differential detergent solubilization yielded CYT, IM, PERI, and OM fractions (Figure 1) from which tryptic peptides were identified using LC-MS/MS (see Section 2). The total number of peptide observations from each protein (spectral count) was used as an estimate of relative abundance, and a protein was considered present in a sample only if observed in at least two of three replicates. This step served the dual purpose of globally removing proteins with only one peptide observation and increasing confidence in peptide identifications within each subcellular fraction. The average sequence coverage for each protein was ~30%. Similar numbers of proteins were identified in LB (993), LPM-4h (1102), and LPM-20h (1006) growth conditions.

### 3.2. Subcellular Fraction Enrichment

 Each subcellular fraction contained a unique protein profile (Supplemental Figure 1), although the IM contained a larger proportion of cofractionating CYT proteins, as noted previously [9]. We avoided high-pH treatment of membrane fractions [27] in an attempt to maintain physiologically relevant protein-protein and protein-membrane interactions; thus, peripheral membrane proteins were not removed in our protocol.

**Figure 2:**
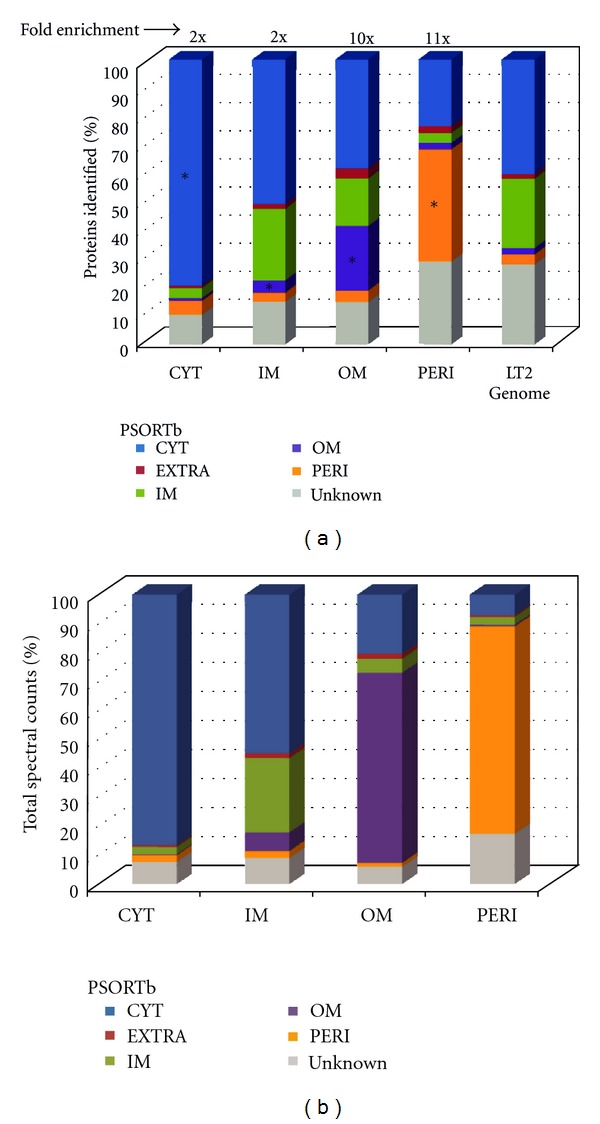
Distribution of proteins observed in subcellular fractions via LC-MS/MS (a). Protein composition of each subcellular fraction, based on number of proteins observed in each fraction sorted according to predicted subcellular location [16]. Data are percentage of proteins observed in each fraction. The fold-enrichment in proteins compared to the genomic potential is noted above each bar. *P ≤ 0.002, *χ* ^2^ test, compared to genome (b). Summed spectral counts (total abundance) of proteins observed in subcellular fractions.

Agreement between observed and computationally predicted protein localization was assessed. Subcellular predictions were computed using PSORTb [28], with the caveat that ~17% of the observed proteins had no PSORTb subcellular assignment (unknown or unknown with multiple possible localizations). Each subcellular fractionation was enriched in the types of proteins expected to reside there (Figure 2(a); Table 1). Both the IM and OM contained a large number of predicted CYT proteins. Since many proteins were likely observed in multiple fractions as minor contaminants due to cofractionation, protein abundance contributions were more informative than the absolute number of proteins observed [9]. From this analysis, predicted CYT proteins contributed to 80–86% of the total protein abundance in CYT fractions, predicted OM proteins, to 65–80% in OM fractions, and predicted PERI proteins, to 68–75% in PERI fractions. In contrast to the expected agreement between predicted and observed enrichment, predicted IM proteins contributed to only ~25% of the total protein abundance observed in IM fractions (Figure 2(b)). This relatively limited enrichment was due largely to cofractionation of abundant CYT proteins and to the general low observability of integral membrane proteins by proteomics [29, 30].

As many proteins involved in bacterial pathogenesis are located outside the cytoplasm where they may more readily target and respond to the host environment, we assessed our success in enriching envelope proteins in the appropriate fractions. Cell envelope (IM, PERI, and OM) proteins can be distinguished by physicochemical properties, such as hydrophobicity (IM proteins), amphipathic beta sheets (OM proteins), and signal peptides (many envelope proteins). The IM, PERI, and OM were significantly enriched in envelope proteins based on observed physicochemical properties. For example, 239 proteins with predicted signal peptides (using PSORTb) were observed (45% of genomic potential). These proteins were mainly identified in the IM, OM, and PERI fractions, with the highest number (130) observed in the PERI (Table 1). Of 51 predicted outer membrane *β*-barrel proteins [31] observed (51% of genomic potential), 44 of these were in outer membrane fractions. Similarly, proteins with predicted transmembrane *α*-helices [32], a feature of integral membrane proteins, were concentrated in the IM, as expected. Of 97 proteins with ≥3 transmembrane domains, all were observed in the IM, while only 24 were observed in the other three fractions combined (Table 1). Hydrophobicity, another hallmark of integral membrane proteins [33], correlated well with proteins observed in IM samples. For the 66 proteins that could be considered very hydrophobic (hydrophobicity average ≥0.5) [33], all were observed in the IM with high abundance values (not shown), while 3, 0, and 17 were observed in the CYT, PERI, and OM, respectively.

### 3.3. Determination of Primary Observed Localization

**Figure 3:**
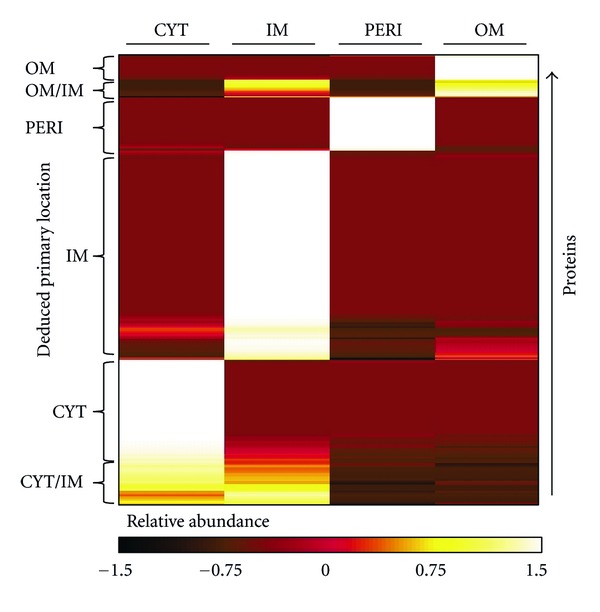
Use of Z-scores and K-means clustering to assign primary subcellular locations to proteins. Since many proteins were observed in two or more subcellular fractions, Z-scores of spectral counts across the four fractions were calculated to highlight the primary observed localization of each protein. K-means clustering was used to group proteins with similar profiles.

For proteins observed in multiple subcellular fractions, it was useful to identify the fraction in which each protein was observed at its highest level (i.e., the likely true subcellular location of the protein). Primary localization was determined within each growth condition by calculating the Z-score of protein abundance in each subcellular fraction. Z-scores were clustered using the K-means algorithm to group similar profiles of subcellular localization (Figure 3; Supplemental Table 3). Note that similar approaches have been described previously [7, 9]. Using the LB culture as an example, 91% of proteins could be assigned a single primary localization using this scheme. 

Some proteins (~9%) were highly observed in two or more subcellular fractions and usually occurred between the CYT and IM or IM and OM. It is noteworthy that six of the 22 IM/OM proteins were lipoproteins, which likely reflects the increased hydrophobicity and tendency to partition with the Sarkosyl-soluble IM. Other members of the IM/OM class included membrane-bound portions of type 3 secretion systems (T3SS): PrgH and PrgK of the invasion-related T3SS and FliF, FliG, and FlgE that represent the ring, basal body, and hook of the flagellar T3SS. In these cases, cofractionation reflects the association of these supramolecular structures with both membranes.

**Figure 4:**
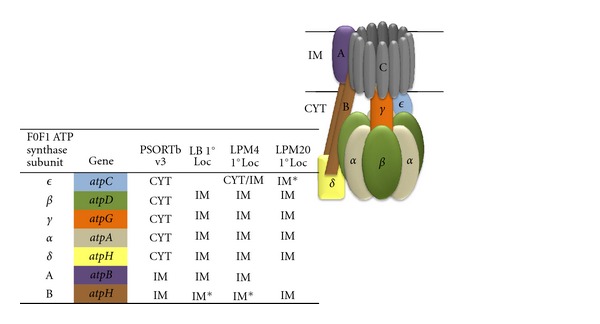
ATP synthase complex exemplifies observed protein-protein and protein-membrane interactions. Schematic representation of membrane-bound ATP synthase, modeled after KEGG Bacterial F-type ATPase Color-coded table shows protein observations in subcellular fractions. n/a: protein not observed. C: AtpE (not observed in this study). *Protein was exclusive to one subcellular fraction in a given growth condition.

Of the proteins that were multilocalized or had secondary locations, several have been implicated in strong physiologically relevant protein-protein and protein-membrane interactions that can influence localization. For example, seven of the eight subunits of ATP synthase were observed primarily in the IM fraction (Figure 4). While only two subunits are integral to the IM, close protein-protein interactions likely mediated the cofractionation of the entire complex to the IM. Peripheral membrane proteins and multisubunit cytoplasmic proteins made up a majority of the known CYT proteins that had IM or IM/CYT as their primary observed location. Using a combination of available subunit information in Uniprot (http://www.uniprot.org/) and published literature, 45 of the 50 IM/CYT proteins were justified in their observed location due to their multimeric forms or peripheral membrane association that are tied to protein function (Supplemental Table 4).

Another group of proteins in this class were the two-component regulatory systems. These systems consist of a membrane-bound sensor-kinase protein and a cytoplasmic response regulator that interacts with, and is phosphorylated by, the sensor-kinase at the membrane, which promotes DNA binding and regulation of gene expression [34]. In both the PhoP/PhoQ and ArcA/ArcB systems, the sensor-kinases were observed exclusively in the IM, while the response regulators were observed either in the IM (i.e., presumably bound to the kinase) or in the CYT (i.e., presumably interacting with DNA), depending on growth condition (Table 2). Our results iterate that PhoP is bound to DNA during growth in LPM (for either 4 or 20 h), which is supported by known activation of the PhoP regulon within acidified macrophage phagosomes [35] and during growth under phagosome-mimicking conditions [26]. Conversely, the response regulator ArcA is IM-localized in cells grown in LB or those grown in LPM for a short duration, but is CYT-localized in cells grown overnight in LPM. These results provide insight into the function of this regulatory system under these specific growth conditions.

We note that some instances of multilocalized proteins may be due to the inability of our methods to perfectly resolve subcellular fractions, or may be artifacts of fractionation. As an example of the latter, DnaK and Ef-Tu can be translocated out of the cytoplasm during osmotic shock [36]. In our study, Ef-Tu was observed at high levels in both the IM and CYT. While DnaK was observed primarily in the CYT, DnaJ, a cochaperone with DnaK, was observed primarily in the IM in all growth conditions in this study.

For those proteins annotated as “putative” (*n* = 274) or “hypothetical” (*n* = 92), we were able to confidently assign localization to a majority based on protein abundances in subcellular fractions (Supplemental Table 5). For many of these proteins, the assignment of subcellular localization as well as data on relative expression levels in different growth conditions represents the most extensive characterization available to date.

### 3.4. Putative Moonlighting Proteins

Some proteins were observed in unexpected subcellular locations regardless of growth condition, while the location of other proteins appeared to be influenced by growth condition. Several proteins with well-characterized housekeeping roles (e.g., enolase and glyceraldehyde-3-phosphate dehydrogenase) have been observed on the cell surfaces of pathogens, where they have secondary functions such as adhesion and immune modulation [3]. The term “moonlighting” refers to proteins that exhibit more than one biological function [1–3]. Here too, proteins that were observed in unexpected locations based on predictions, annotations, and known functions could point to novel interactions or functions yet to be characterized. In these cases, proteins with higher spectral counts (relative abundance) and greater numbers of unique peptides (more confident identifications) were considered more reliable candidates for assignment of localization.

**Figure 5:**
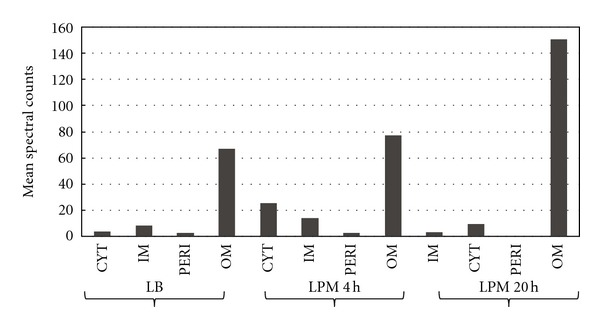
Localization and relative abundance of potential moonlighting protein, Dps. Spectral counts of Dps in each subcellular fraction in each growth condition. Values are means of 3 replicates.

One of the best moonlighting protein candidates observed in this study is Dps (DNA protection during starvation). This protein has been well characterized as a cytoplasmic DNA-binding protein (reviewed in [37]) and has no predicted signal peptide. In each growth condition tested, we observed Dps significantly enriched in the OM fraction (Figure 5), which shows for the first time that this protein is OM-localized in *Salmonella*. Dps is a known virulence determinant of *Salmonella* [38], but how it translocates to the OM and its role(s) at the cell surface remain to be investigated. Interestingly, Dps was recently observed on the cell surface of *Escherichia coli* [38, 39], where it may play a role in attachment to abiotic surfaces [38]. We observed >2-fold increase in the relative abundance of OM-localized Dps between LB and LPM20 growth conditions, which indicates that *Salmonella* Dps is responsive to growth under phagosome-mimicking conditions (Figure 5).

Because the CYT and OM are the two most physically separated subcellular locations studied here and contain proteins with fairly distinct physicochemical properties, we considered known cytoplasmic proteins observed in the OM as the most promising moonlighting candidates. These candidates included a (3R)-hydroxymyristoyl-ACP dehydratase (FabZ), a curved DNA-binding protein (CbpA), an imidazole glycerol-phosphate dehydratase/histidinol phosphatase (HisB), and an ATP-dependent RNA helicase (SrmB). All of these cases included proteins generally accepted to be cytoplasmic, with no detectable signal peptides, transmembrane helices, or beta barrel predictions that were confidently observed in OM or in a mix of OM and IM fractions (Supplemental Table 6). These proteins represent the first candidates for an investigation of moonlighting activities in *Salmonella*.

### 3.5. Subcellular Responses to Growth Conditions

 Although not a perfect replica of the *in vivo* environment, defined *in vitro* synthetic growth media provide valuable insights into the pathogenic strategies of *Salmonella* [40, 41]. Growth in LB to mid-exponential phase induces genes of the *Salmonella* pathogenicity island 1 (SPI-1) involved in host cell invasion [42–44], while genes of the *Salmonella* pathogenicity island 2 (SPI-2) can be induced by growth in LPM that simulates the environment of the *Salmonella*-containing vacuole (phagosome) [45, 46]. We used these growth conditions to probe the subcellular-level responses of *Salmonella* to phagosome-mimicking conditions.

When qualitatively assessed, similar numbers of proteins were observed in the three growth conditions: 993 in LB, 1102 in LPM-4h, and 1006 in LPM-20h. Approximately 10% of the proteins identified in each growth condition were unique to a given culture: 175 in LB, 100 in LPM-4h, and 92 in LPM-20h (Supplemental Figure 2), and less than half of all identified proteins (688) were observed in all growth conditions, which underscores the utility of using multiple growth conditions for improved coverage of a bacterial proteome.

We have previously investigated the proteome response of *Salmonella* to phagosome-mimicking *in vitro* conditions [19, 26]; however, the use of subcellular fractionation presented an opportunity for obtaining better proteome coverage, especially of proteins that are typically underrepresented in global proteomic strategies, in addition to highlighting the subcellular location of proteins of interest. Based on studies of *Salmonella* grown in acidic minimal media [19, 26], we confirmed the expected increases in abundance of proteins associated with the SPI-2 T3SS (SsaC, SseA, and SsaJ), the SsrB regulon (SsrA, SsrB, and SrfN), and the PhoP regulon (PhoP, PhoQ, PagC, MgtA, and MgtB) during growth in LPM (Supplemental Table 7). Conversely, proteins related to the invasion-associated SPI-1 T3SS (SipA, B, C, D, SopB, SicA, InvG, PrgK, and PrgL) decreased in abundance with growth in LPM. Further analyses focused on envelope proteins because the proteins primarily detected in previous global analyses were cytoplasmic proteins and because envelope proteins have high potential for host-pathogen interactions.

**Figure 6:**
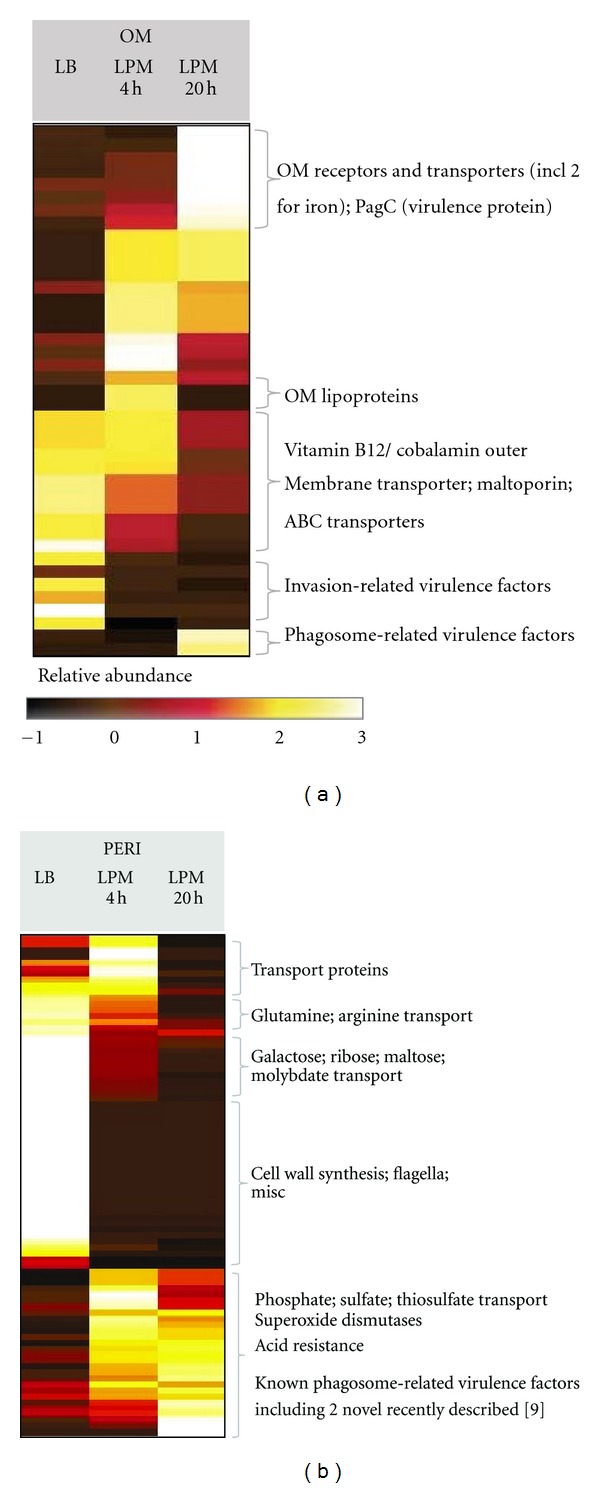
Heat map representation of differentially expressed OM and PERI proteins. Z-scores of protein abundance were calculated across the 3 growth conditions for proteins observed at their highest levels in the OM (a) and PERI (b) fractions. Each protein showed ≥ 2-fold difference in abundance in any two growth conditions.

OM proteins whose abundance increased during growth in LPM included iron transporters (FepA, FhuA, IroN, and FoxA), ABC transporters, and virulence-related proteins (PagC; T3SS-related SsaC and SseC), which reflects the nutrient-limited and virulence gene-inducing nature of LPM (Figure 6(a)). A notable OM protein was the outer membrane protease PgtE that was increased 13- and 89-fold in LPM4 and LPM20, respectively (*P* < 0.001). PgtE is involved in cleavage of serum complement during the extracellular phase of *Salmonella* systemic infection [47], but its induction under phagosome-mimicking conditions suggests an intracellular role as well. In addition to the importance of OM proteins that increase in abundance in LPM, those that decrease in abundance may be indicative of immune evasion or virulence-related OM remodeling. For example, putative outer membrane lipoprotein maltoporin and outer membrane protein N were significantly decreased during growth in LPM for 20 h (Supplemental Table 7). Known SPI-1 T3SS-related surface proteins such as PrgK, PrgI, and InvG were also significantly decreased in the OM during growth in LPM, indicating the expected shift away from SPI-1 T3SS expression during growth in LPM.

Notable in the IM was a decrease in chemotaxis-related proteins (CheA, B, M, and Z; Tsr, Trg, and Tcp) and motility-related proteins (FliF, FliI, FliN, and MotA) in LPM compared to LB. A range of IM-integral and peripheral IM proteins of various functions were enriched during growth in LPM, including expected functions such as magnesium transport (MgtA and MgtB), virulence proteins (PhoQ and SsaC), and various transporters, enzymes, and proteins of unknown function (Supplemental Table 7).

The PERI shifted from transport of sugars (galactose, ribose, and maltose), oligopeptides, dipeptides, aminoacids, and related compounds (arginine and putrescine) in LB to transport of phosphate, sulfate, and thiosulfate in LPM (Figure 6(b)). Also showing increased abundance in LPM were PERI proteins involved in superoxide and acid resistance (SodC and PhoN) and known secreted factors CigR [14] and SrfN [48, 49] for which the subcellular location prior to being translocated into infected mammalian cells was previously unknown.

## 4. Discussion

Comparative proteomics is an emerging tool for studying bacterial pathogenesis both* in vitro* and during infection [19, 26, 50]. Subcellular fractionation complements such analyses by providing a means to resolve physiologically relevant protein location in the bacterium. Our analysis of CYT, IM, PERI, and OM fractions of *S. Typhimurium* grown under laboratory and phagosome-mimicking conditions yielded ~1400 unique proteins, most of which could be confidently localized to a single subcellular fraction in a given growth condition. Each subcellular fraction contained a unique protein profile (Figures 1 and 3 and Supplemental Figure 1) and protein physicochemical properties generally agreed well with their observed localization (Table 1).

To our knowledge, this study represents the most comprehensive global survey of subcellular localization in *Salmonella* to date. In earlier work, Coldham and Woodward [51] assessed cytosolic, cell envelope, and outer membrane protein preparations of *Salmonella* by extensive chromatographic fractionation followed by mass spectrometry. They observed 816 proteins, with 371 in the CYT, 565 in the envelope, and 262 in the OM samples. Of the latter 262, only 20 were OM proteins. Recently, the OM proteome of *S. enterica* was identified using a lipid-based method [52]. In that study, 54 OM proteins were identified with ≥2 peptides, using a multistep digest procedure on outer membrane vesicle preparations. In an early attempt to catalogue the OM proteome of *Escherichia coli*, Molloy and colleagues [27] identified ~30 proteins in the OM fraction, using 2D gel electrophoresis and MS approach. In our present study, at least 74 OM proteins were identified in OM fractions (deduced by PSORTb prediction, annotation, or by the presence of OM *β*-sheets). In addition to high coverage of OM proteins, confident assignment of CYT, IM, and PERI proteins was presented (Supplemental Table 3).

Among the challenges in any subcellular fractionation endeavor are to maximize fraction purity and correctly assign proteins to a subcellular location. Due to the close proximity of fractions, protein-protein interactions between fractions, or to the presence of protein domains that span multiple fractions, proteins sometimes copurify to two or more fractions. These biological phenomena are difficult to distinguish from experimental noise. In our analysis, large multi-subunit cytoplasmic complexes often concentrated in the membrane fractions (particularly the IM); likewise, many protein complexes that are known to be peripherally IM-associated also co-fractionated with the IM (e.g., ATP synthase). In cases where a protein was observed in multiple fractions, we were able to use relative abundance data to deduce the primary observed localization (Figure 3). However, some fractions posed more of a challenge than others; for example, the IM was more ambiguous than the OM, PERI, or CYT. Over 40% of proteins whose primary observed location was the IM were predicted by PSORTb to be cytoplasmic. It is important to note that this localization prediction does not take into account the many potential IM-interacting proteins. While the IM fraction is a good potential source of novel protein-protein and protein-membrane interactions, a clearer picture of the integral IM landscape could emerge upon high-pH buffer treatment of the IM fraction to remove peripherally bound proteins [27].

An aspect of this study that may be helpful to others interested in subcellular proteome characterization was our use of a mutant that was depleted in an abundant cell envelope component, flagellin (Δ*fliC*Δ*fljB*). Because flagellin was one of the most abundant proteins observed in the PERI (and contaminated all envelope fractions) in a preliminary subcellular proteomic analysis (Supplemental Table 1), we hypothesized that deleting flagellin genes would enable better detection of low abundance of PERI proteins and likely increase the signal of most other proteins in the PERI fraction. Flagella are not essential for survival in macrophage phagosomes [53] and are downregulated under the environmental conditions simulated by our mLPM culture condition [20]. Thus, deleting flagellins should not interfere with the physiological responses we were interested in. In addition, flagella are nonessential for growth in LB (not shown). Proteomic analysis of the wild type versus Δ*fliC*Δ*fljB* mutant PERI fractions showed no differences in presence of “housekeeping” proteins such as elongation factor Tu, elongation factor G, chaperonin GroEL, and ribosomal proteins that co-fractionated with the PERI (Supplemental Table 2). Also, IM and OM proteins that co-fractionated with the PERI were observed at similar (low) levels in both the wild type and mutant. Most importantly, we observed higher spectral counts of PERI proteins in the mutant relative to wild type, and several PERI proteins were detected only in the flagellin mutant (Supplemental Table 2). Thus, we advocate the use of relevant mutations in abundant nonessential proteins for improved subcellular proteome coverage.

The availability of experimentally observed subcellular localization data for such a large number of *Salmonella* proteins provides opportunities for further study. Among these opportunities are using high-confidence localization information for training subcellular localization prediction tools and for computationally predicting *Salmonella* function in host cells through the use of genome-scale models [4]. In addition, localization data for hypothetical or uncharacterized proteins (Supplemental Table 5) is a first step towards functional characterization of these unknown proteins. To extend the utility of these data, our future study will focus on multilocalized proteins and those that changed localization depending on growth condition. Both categories present the possibility of exciting discoveries in terms of protein function. Moonlighting protein candidates are included in this class; determining the transport mechanism and secondary function of our candidates are challenges for future study.

In summary, we presented a comparative subcellular proteomic analysis of *Salmonella *representative of laboratory growth and infection-like states. We cataloged the confident localization of over 1000 proteins and provided evidence of differential protein movement and the appearance of some proteins in unexpected subcellular compartments. These results imply the existence of unknown transport mechanisms and novel functions for a subset of *Salmonella* proteins.

## Supplementary Material

Supplemental Figure 1 shows a 1D SDS-PAGE analysis of subcellular fractions of WT and flagellin mutant. Supplemental Figure 2 is a Venn Diagram illustrating the overlap in protein identifications among the three growth conditions. Seven supplemental data tables provided include (1) a preliminary MS analysis of Salmonella subcellular fractions and rationale for flagellin mutants, (2) a comparison of the PERI fractions in WT versus flagellin mutant, (3) K-means clustering of Z-transformed protein abundances to determine primary protein localization, (4) analysis of IM-colocalized CYT proteins, (5) subcellular localization of hypothetical and uncharacterized proteins, (6) moonlighting candidate proteins, and (7) analysis of protein abundance changes under different growth conditions.

## Figures and Tables

**Table 1:** Enrichment of proteins with expected physicochemical properties.

Protein type	CYT	IM	PERI	OM	All observed	In genome	Percentage observed
OM beta barrel	8	27	7	**44**	51	99	52%
Signal Peps	81	120	**130**	100	239	532	45%
TMD > 0	26	**196**	6	54	204	1167	18%
TMD > 1	10	**130**	2	33	130	812	16%
TMD > 2	4	**97**	2	21	97	683	14%
TMD > 3	3	**88**	2	19	88	619	14%
GRAVY > 0	258	**488**	54	140	611	2882	21%
GRAVY > 0.1	158	**335**	31	88	413	2201	19%
GRAVY > 0.2	69	**194**	16	47	231	1637	14%
GRAVY > 0.3	24	**133**	8	34	145	1276	11%
GRAVY ≥ 0.5	3	**66**	0	17	66	890	7%

**Table 2:** Two-component regulators showing localization changes depending on growth conditions.

Protein Description (PhoP/Q)	Gene	PSORTb v3	LB 1°Loc	LPM4 1°Loc	LPM20 1°Loc
Sensor protein PhoQ	PhoQ	IM	IM^∗^	IM^∗^	IM^∗^
DNA-binding transcriptional regulator PhoP	PhoP	Cyt	IM/CYT	CYT	CYT

Protein Description (ArcA/B)	Gene	PSORTb v3	LB 1°Loc	LPM4 1°Loc	LPM20 1°Loc

Aerobic respiration control sensor protein ArcB	ArcB	IM	IM^∗^	IM^∗^	IM^∗^
Two-component response regulator	ArcA	Cyt	IM^∗^	IM	CYT

^∗^Indicates that a protein is observed exclusively in one location.

## Abbreviations

IM: Inner membrane
OM: Outer membrane
CYT: Cytoplasmic
PERI: Periplasmic
WCL: Whole cell lysate
LC-MS/MS: Liquid chromatography-tandem mass spectrometry
TTSS: Type III secretion system
SPI: *Sa* *lm* *on* *el* *la* pathogenicity island.
