# Supplementary figures and images for: A Comprehensive Subcellular Proteomic Survey of Salmonella Grown under Phagosome-Mimicking versus Standard Laboratory Conditions
Source: Int J Proteomics. 2012 Jul 25;2012:123076. doi: 10.1155/2012/123076 (PMC3410353; doi:10.1155/2012/123076)

**WT**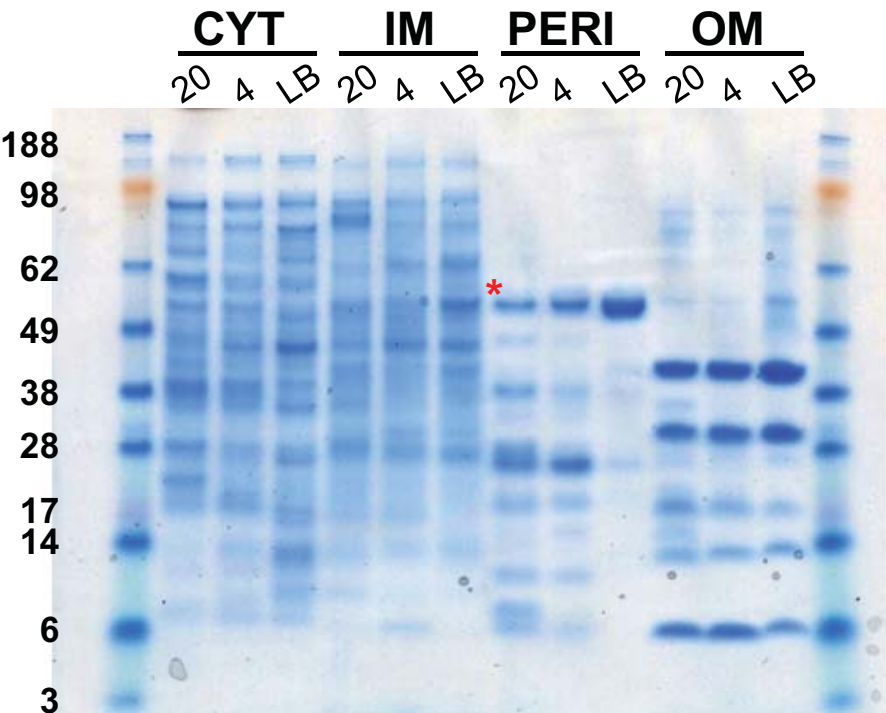 **$\Delta fliC/\Delta fliJ$** 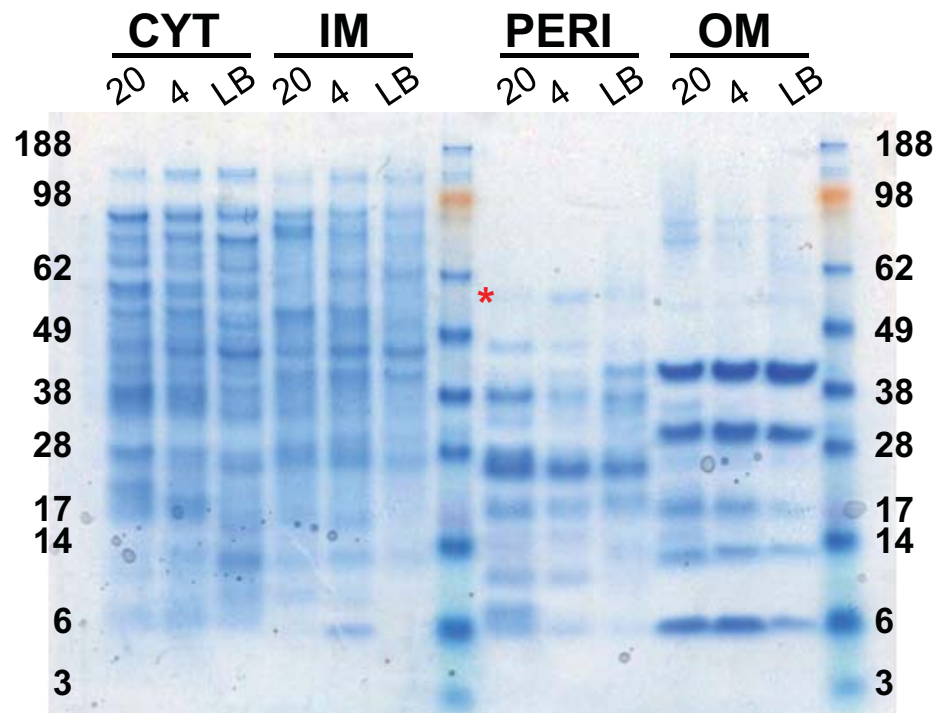

Supplement: Supplementary file 1 — Supplemental Figure 1 shows a 1D SDS-PAGE analysis of subcellular fractions of WT and flagellin mutant. Supplemental Figure 2 is a Venn Diagram illustrating the overlap in protein identifications among the three growth conditions. Seven supplemental data tables provided include (1) a preliminary MS analysis of Salmonella subcellular fractions and rationale for flagellin mutants, (2) a comparison of the PERI fractions in WT versus flagellin mutant, (3) K-means clustering of Z-transformed protein abundances to determine primary protein localization, (4) analysis of IM-colocalized CYT proteins, (5) subcellular localization of hypothetical and uncharacterized proteins, (6) moonlighting candidate proteins, and (7) analysis of protein abundance changes under different growth conditions. [file 123076.f1.pdf]

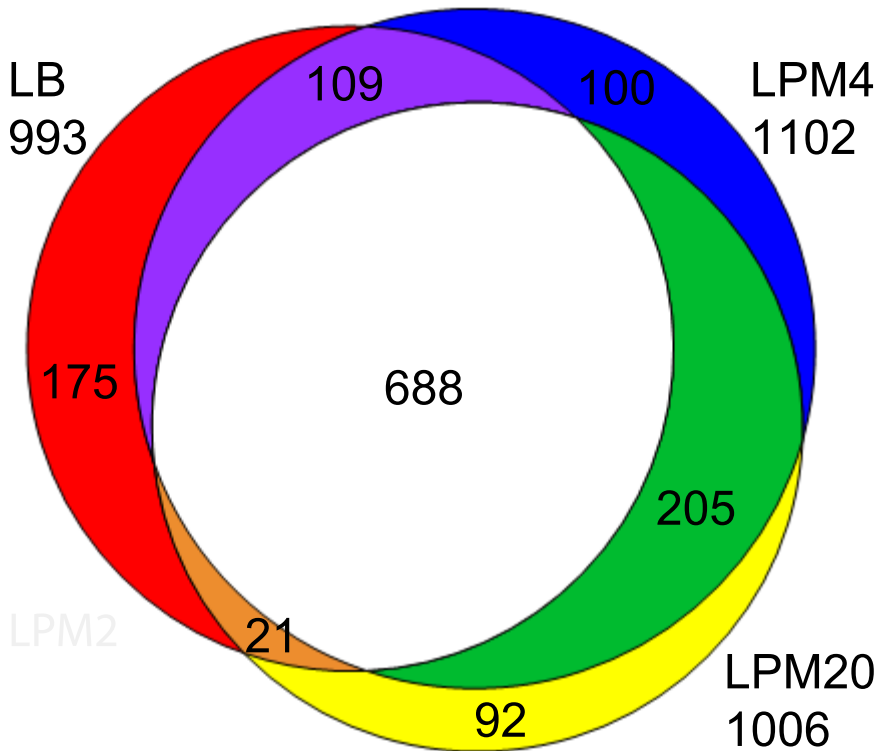

Supplement: Supplementary file 2 [file 123076.f2.pdf]
